# The Year in Cardiology 2015: Arrhythmias and Device Therapy

**DOI:** 10.1093/eurheartj/ehv725

**Published:** 2016-11

**Authors:** Jan Steffel, Pierre Jais, Gerhard Hindricks

**Affiliations:** 1 Cardiac Arrhythmia Unit - Department of Cardiology - University Hospital Zurich, Zürich, Switzerland; 2 Hôpital Haut-l'évêque - Departments of Cardiology and Radiology - Centre Hospitalier Universitaire (CHU) de Bordeaux & LIRYC Institute - Institut Hospitalo-Universitaire (IHU), Zürich, Switzerland; 3 Bordeaux, France; Department of Electrophysiology - University of Leipzig - Heart Center, Leipzig, Germany

**Keywords:** Arrhythmias, Atrial Fibrillation / diagnosis, Catheter Ablation / trends, Catheter Ablation / methods, Defibrillators Implantable / trends.

## Preamble

The year 2015 was once more filled with exciting and important novel developments in the field of invasive electrophysiology and implantable cardiac devices. These include technical innovation, novel molecular and cellular insights, and presentation of large randomized clinical trials as well as important 'real-world' registries. In addition, several new guidelines surfaced in 2015, including those for the treatment of ventricular arrhythmias and prevention of sudden cardiac death. It is virtually impossible to cover all novel developments that would merit discussion in this type of overview; as a result, the authors had to make a selection, focusing on several important developments with direct implications for daily clinical practice.

## Cardiac arrhythmias and catheter ablation

### Atrial fibrillation

Catheter ablation of atrial fibrillation (AF) remained in focus of clinical studies and large-scale trials. The use of force-sensing ablation catheter technologies seems to improve the induction of durable atrial lesions and was shown to significantly reduce AF recurrence rate after catheter ablation in a meta-analysis mainly made of non-randomized trials.^1^ This technology will become standard for AF catheter ablation in the future. A word of caution: there is growing evidence that more extensive ablation in the atria does not *per se* improve the rhythm outcome after AF catheter ablation. The Minimax Trial compared two ablation strategies for pulmonary vein isolation (PVI) in 234 patients who underwent catheter ablation of paroxysmal AF: circumferential antral PVI alone ('minimal') vs. PVI with intravenous ridge ablation to achieve individual PVI ('maximal'). After a mean follow-up of 17 ± 8 months, freedom from AF after limited 'minimal' ablation was not worse compared with more extensive 'maximal' ablation (70 vs. 62%; p = 0.25).^2^ Previous data indicated that adenosine-guided detection of dormant pulmonary vein re-conduction and subsequent re-isolation of the veins can be successfully applied to improve outcome of AF catheter ablation.^3^ However, a much bigger randomized trial published in *European Heart Journal* now questioned the usefulness of adenosine testing: in the Japanese UNDER anti-tachycardia pacing (ATP) Trial, 2113 patients were randomized to either adenosine challenge or control and no difference in AF recurrence rate was shown at 1 year.^4^ The reasons for the contradictory results reported from these two multi-centre, randomized trials are unclear at present and deserve further investigation. Treatment with anti-arrhythmic drugs after catheter ablation was shown to reduce the AF recurrence 90 days after catheter ablation in the EAST AF trial, however, at 1 year there was no difference in arrhythmia recurrence between treatment and control group.^5^ These results are quite in line with the data of the AmioCat Trial.^6^ In AmioCat patients were randomized to amiodarone or placebo for 8 weeks after AF catheter ablation. While amiodarone treatment reduced hospitalizations and cardioversions in the 3-month post-ablation blanking period, there was no difference in AF recurrence rate at 6-month follow-up (39 vs., 48%; p = 0.18). Thus, anti-arrhythmic drugs may prevent early AF recurrences after ablation but may not promote a better atrial re-modelling resulting in a higher sinus rhythm rate during follow-up. The 5-year follow-up data of the MANTRA-PAF Trial were reported during the ESC Congress in London: MANTRA-PAF evaluated the comparative effects of first-line radiofrequency catheter ablation of AF with anti-arrhythmic drug therapy. At 2-year follow-up, there was no difference in cumulative AF burden between the ablation and anti-arrhythmic drug group, while the burden of AF was significantly lower in the ablation group (90th percentile, 9 vs. 18%; p = 0.007).^7^ However, at 5-year follow-up, there was a significantly higher rate of AF-free patients in the ablation compared the anti-arrhythmic drug treatment group (86 vs. 71%; p = 0.001). Also, AF burden was lower in the ablation compared with the drug group (p = 0.003). Interestingly, the effects on quality of life were similar in both groups. These data indicate that the rhythm benefit resulting from catheter ablation may increase over time; however, it is important to understand that MANTRA-PAF was too small to evaluate any effect of ablation or anti-arrhythmic drugs on hard outcome parameters such as stroke and/or mortality. These questions will be open until data from the EAST Trial (endpoint: composite of death, stroke, and heart failure) and CABANA Trial (endpoint: composite of death, serious bleeding, disabling stroke, and cardiac arrest) are available.^8,9^ Persistent AF ablation strategy has never been mature enough for a consensus to emerge, neither in the past nor in 2015. Rotor ablation using contact phase mapping has been questioned,^10^ and CAFÉ ablation is not specific enough to be convincing as demonstrated by a large meta-analysis.^11^ In contrast, lifestyle modification such as weight loss is remarkably effective in reducing AF burden (10% loss translates into a six-fold AF burden reduction) and in inducing reverse remodelling on left atrial size and left ventricular septal thickness.^12^

#### Stroke prevention

Due to the results from large-scale clinical trials, the non-vitamin K antagonist oral anticoagulants (NOACs) are the preferred treatment for stroke prevention in non-valvular AF, as reflected in current ESC guidelines.^13^ As the fourth NOAC, edoxaban has been approved in 2015 in many countries including the USA, Switzerland, and Europe based on the results of the ENGAGE AF-TIMI 48 trial.^14^ During the year 2015, several subgroup analyses of the large NOAC trials have surfaced, including bleeding management and outcome with apixaban,^15^ the management of rivaroxaban around catheter ablation for AF (VENTURE-AF),^16^ and the outcome of amiodarone co-medication in patients receiving edoxaban,^17^ to name just a few. Virtually, all subgroups of the large NOAC trials indicate a consistent benefit and safety of these drugs compared with warfarin, further underlining their overall superiority. This is supported by important real-world data (including those from a prospective registry with rivaroxaban, XANTUS)^18^ indicating efficacy and safety, which is in line with that observed in the randomized clinical trials.

Arguably, the most exciting novelty in the field of NOACs comes from the development of specific reversal agents ('antidotes'). In a Phase 1 study in healthy men, the monoclonal antibody idarucizumab (specific for dabigatran) was well tolerated with no unexpected or clinically relevant safety concerns, and was associated with immediate, complete, and sustained reversal of dabigatran-induced anticoagulation.^19^ Moreover, in a Phase 3 study, idarucizumab was demonstrated to effectively and immediately reverse the anticoagulant effect of dabigatran in patients presenting with serious bleeding or requiring an urgent procedure.^20^ As a result, the US Food and Drug Administration has approved the drug in October 2015; the Committee for Medicinal Products for Human Use of the European Medicines Agency has also recently issued a positive opinion, and approval is expected by the end of this year or early 2016. Importantly, idarucizumab is ineffective against Xa-inhibitors; instead, different directly acting antidotes are being developed, including andexanet alfa and PER977. First results are also positive with these agents, and larger-scale clinical trials are anticipated within the year 2016. While these drugs clearly represent an important addition to our portfolio, many aspects in the practical use remain to be determined, including the type of patients and conditions requiring reversal and the time of reinstitution of anticoagulation. These and other issues are elegantly described in the 2015 updated version of the European Heart Rhythm Association practical guide,^21^ following the great success of its first version published in 2013.^22^

Will catheter ablation of AF have an impact on stroke risk? Novel data from a large Danish registry suggest a very low risk of stroke for patients after catheter ablation.^23^ However, these data do require validation in a prospective randomized trial before clinical practice for oral anticoagulation after catheter ablation may be changed.^24^

#### Ventricular arrhythmias and sudden cardiac death

**Figure 1:**
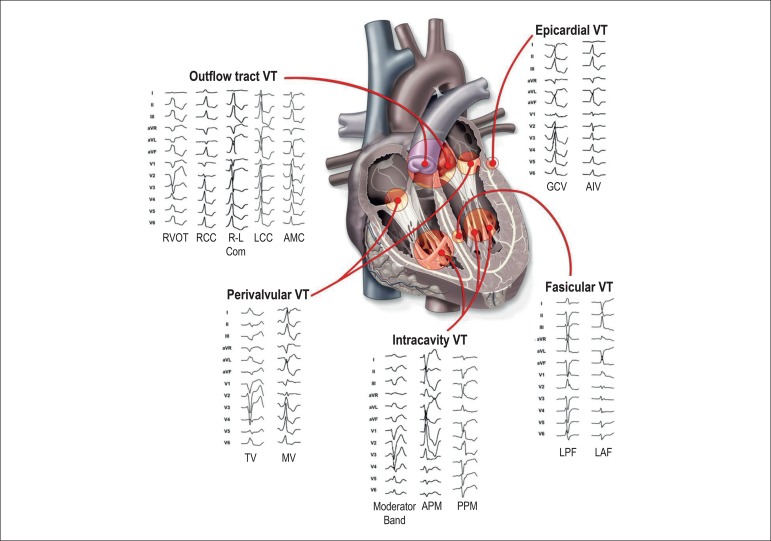
Twelve-lead electrocardiogram morphology of different sites of origin in idiopathic ventricular tachycardia. RVOT: right ventricular outflow tract; RCC: right coronary cusp; R–L com: right–left coronary cusp commissure; LCC: left coronary cusp; AMC: aortomitral continuity; TV: tricuspid annulus; MV: mitral annulus; APM: anterior PAP; PPM: posterior PAP; LPF: left posterior fascicle; LAF: left anterior fascicle; GCV: greater cardiac vein; AIV: anterior inter-ventricular vein. Reproduced from Tanawuttiwat et al.,^25^ reprinted with kind permission from Tanawuttiwat et al.^25^ This Figure has been reprinted by permission of Oxford University Press on behalf of the European Society of Cardiology.

Catheter ablation of ventricular tachycardia (VT) is one of the fastest growing fields in interventional electrophysiology^25^; the importance of diagnosing and correctly triaging VTs, particularly those easily amenable to catheter ablation (Figure 1), is a challenge faced by cardiologists on a regular basis. Multiple important studies have been reported within the last 12 months documenting the importance and increased utilization of VT ablation. Despite several remarkable technical and technological improvements and innovations such as use of image integration,^26^ novel ablation electrodes,^27,28^ force-sensing technologies,^29^ or ultra-high density mapping,^30^ the relatively high recurrence rate of any VT after catheter ablation in patients with VT and structural heart disease remains a key challenge. As evident from recent multi-centre data, non-inducibility of any VT at the end of the ablation is probably the best endpoint for the procedure and should be targeted.^31^ In addition, non-inducibility when supported by elimination of abnormal potentials may also have an impact on survival as well.^32,33^ Most fascinating is the report of successful 'ablation' of Brugada syndrome. The idea to treat Brugada patients at risk of sudden cardiac death with an interventional ablation procedure is further advanced by a recent report from Brugada et al.^34^ In their series, 13 patients underwent epicardial mapping and right ventricular abnormal electrograms were identified in all of them. Catheter ablation normalized the ECG and abolished pre-existing typical ECG changes induced by flecainide. However, despite all enthusiasm, it is unclear whether or not these ablation effects have an impact on spontaneous VT/ventricular fibrillation (VF) and/or risk of sudden cardiac death. The new ESC Guidelines for the treatment of ventricular arrhythmias and prevention of sudden cardiac death were presented during the ESC congress in London.^35^ These guidelines provide up-to-date state-of-the-art summary of current knowledge and best practice treatment in this field.

### Cardiac electronic devices

#### Leadless pacemakers

One of the main trends for cardiac devices in the year 2015 was the continued movement towards the abandonment of intravascular leads. After an initially tedious start, leadless single-chamber pacemakers have finally arrived in daily clinical practice. Early results from the 140 patients receiving the Medtronic MICRA leadless pacemaker system demonstrated a favourable efficacy and safety profile.^36^ During a mean follow-up of 1.9 ± 1.8 months (i.e. covering primarily the perioperative and early postoperative period), no unanticipated serious adverse device events were observed, including no device dislodgement and only one pericardial effusion without tamponade (resulting in prolonged hospitalization). Of note, the latter occurred in a patient in whom the device needed to be repeatedly repositioned (18×). In the majority of patients (81%), however, the device was properly placed with no or only one repositioning. During follow-up, electrical values including pacing thresholds, impedance, and sensing remained stable and favourable, resulting in an anticipated battery longevity of 12.6 years (range 8.6-14.4).^36^ As a result of these findings, the MICRA system received CE mark in the summer of 2015, followed by careful rollout to selected centres and operators after undergoing comprehensive in vivo and ex vivo training. These positive initial results were mirrored in a larger group of 725 patients, of whom 719 (99.2%) underwent successful implantation.^37^ Electrical values (threshold, sensing, and impedance) were favourable in 292 of 297 patients with paired 6-month data. There were 28 major complications in 25 of 725 patients [4.0%, including 11 (1.9%) traumatic cardiac perforation or effusion and 1 death (0.1%)]. These numbers compared favourably with historic controls undergoing transvenous pacemaker implantation. Importantly, no device dislodgements were observed.^37^ Results of the second available single-chamber transcatheter pacing system, the Nanostim (St Jude Medical), were equally presented and published this year.^38^ In the first 526 patients undergoing implantation, the system was successfully implanted in 504 (95.8%). Of the 300 patients who completed 6-month follow-up, the primary efficacy outcome (acceptable electrical values) was reached in 90%. Of the total cohort of 526 patients, serious device-related adverse events occurred in 6.5% of patients, including cardiac tamponade in 5 (1.0%), device dislodgement in 6 (in 1.5%), and device migration during implantation owing to inadequate fixation in 2 patients (0.4%). Further experience with both leadless pacing systems will show how they compare in even larger populations and in daily clinical practice.

Patients with a typical single-chamber pacemaker indication currently represent the primary population for leadless pacers, i.e. permanent AF with symptomatic bradycardia and/or AV block. Future studies and real-world experience will show how these device behave long term (including the novel rate-adaptive sensor system); first personal experiences are encouraging. The development for more advanced systems is ongoing, including dual-chamber pacemakers, cardiac resynchronization therapy, and communication with the subcutaneous implantable cardioverter defibrillator (ICD).

#### Implantable cardioverter defibrillator therapy and implant-based telemonitoring

Implantable cardioverter defibrillator testing is no longer necessary during routine and uncomplicated ICD implantation: in the Nordic ICD Trial, 1077 patients were randomly assigned to first time ICD implantation with (n = 540) or without (n = 537) testing of defibrillation threshold.^39^ Defibrillation efficacy was not different between both groups during follow-up. Similarly, in the SIMPLE trial of 2500 patients, routine defibrillation testing did not result in a reduction in arrhythmic deaths during a mean follow-up of 3.1 years.^40^

Almost all pacemakers and defibrillators that are currently available have the technical option for remote monitoring.^41^ Previous results from randomized clinical trials and analysis from big data sets indicated that these technologies may have beneficial effects when applied appropriately.^42^ However, recent data from the Optilink HF Trial reported at the ESC Congress in London showed disappointing results: the trial randomized 1002 patients with heart failure and an indication for ICD implantation to remote automated pulmonary congestion alert 'on' (n = 505) or 'off' (n = 497). After 18 months of follow-up, there was no significant difference between groups in primary endpoint, which was a composite of all-cause death and cardiovascular hospitalizations. More promising data are derived from the follow-up report of the CHAMPION Trial that assessed the efficacy of automatic pulmonary pressure measurement in heart failure patients to guide and optimize heart failure therapy.^43^ The superiority of the treatment group over the control group previously reported was maintained for an additional 13 months to the end of the Randomized Access Period with a significant reduction of heart failure-related hospitalizations by 33% and of all-cause hospitalizations by 16%. Second, the good results in the treatment group were maintained during an Open Access Period of another 12 months, during which no increase in hospitalizations was observed. Most importantly, heart failure-related hospitalizations and all-cause hospitalizations in the former control group were reduced significantly by 48 and 21%, respectively, after pulmonary artery pressure information became available to guide therapy during the Open Access Period. Thus, implant-based remote telemonitoring seems highly promising to support heart failure therapy and it will be just a matter of time when haemodynamic sensors will be combined with pacemakers, defibrillators, and cardiac resynchronization devices.

#### Subcutaneous implantable cardioverter defibrillators

Ever since its approval in 2009, the subcutaneous ICD (S-ICD) system has increasingly gained attention and attraction. Indeed, its complete lack of intravascularly placed electrodes is potentially associated with a substantial reduction in morbidity (and mortality) due to lead complications associated with currently used 'classical' transvenous ICD systems. In 2015, the new generation EMBLEM S-ICD System was approved, the main feature of which is its 20% thinner size combined with a 40% longer life expectancy when compared with the previous S-ICD system. At the same time, novel algorithms are being developed to overcome the risk of inadequate shock deliveries.^44,45^ Recently published registry results have indicated a decreasing risk of complications, suboptimal programming, and (to a lesser degree) inadequate shock deliveries with increasing experience and volume.^46^ In addition, the same registries demonstrated a high efficacy for the termination of VT and VF, with 90.1% of events (100/111) terminated with one shock and 98.5% (109/111) terminated within the available five shocks.^47^ As a result of these favourable data, the use of the S-ICD has, for the first time, been incorporated into the guidelines for the prevention of sudden cardiac death as a IIa indication [level of evidence (LoE) C] as an alternative to standard ICD for patients without an indication for bradycardia pacing, cardiac resynchronization, or ATP.^35^ Also, the S-ICD may be considered (IIb, LoE C) in patients with difficult venous access, after the transvenous ICD removal for infections or in young patients with a long-term indication for ICD therapy.^35^ Indeed, the lack of possibility to deliver ATP or bradycardia pacing remains the most important shortcoming of current S-ICD devices. Combination of the S-ICD with leadless pacers clearly would be one of the most obvious possible solution to this problem. However, with evidence-based programming (high-rate or long-duration detection zones), the overall amount of delivered ATP will likely be decreasing as a result of both spontaneous VT termination and VTs occurring below the detection limit. A prospective, randomized trial (PRAETORIAN) comparing currently available transvenous and subcutaneous ICDs (i.e. without the possibility of ATP) has been initiated and is currently ongoing.

#### Wearable cardioverter defibrillator

**Figure 2:**
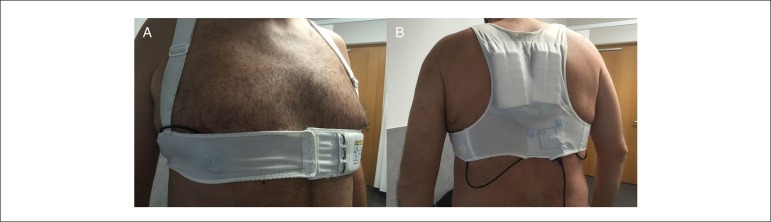
Wearable cardioverter defibrillator. Model of a wearable cardioverter defibrillator (images courtesy of J.S., reprinted with kind permission and patient consent). This Figure has been reprinted by permission of Oxford University Press on behalf of the European Society of Cardiology.

Also for the first time, the new 2015 guidelines for the prevention of sudden cardiac death give recommendations for the use of the wearable cardioverter defibrillator (WCD; Figure 2). With a class IIa recommendation (LoE C), WCD be considered for a limited time period for patients with reduced EF who are at risk of sudden arrhythmic death, but who currently cannot receive an ICD, including patients post-lead removal for infection, patients with active myocarditis, and patients with arrhythmias in the early post-myocardial infarction phase.^35^ In the absence of a randomized clinical trial, this recommendation was based mainly on large registries such as the recently published prospective registry of patients using the wearable defibrillator (WEARIT-II), which followed 2000 recipients of the WCD with a median wear time for 90 days.^48^ In this registry, a total of 120 sustained ventricular tachyarrhythmias (VT/VF) were observed in 41 patients. Of these patients, 54% received appropriate WCD shocks, while only 10 patients (0.5%) received inappropriate WCD therapy.

Importantly, at the end of the individual time frame of WCD use, an ICD was implanted in only 840 patients (42%), with an improvement in EF being the most frequent reason for withholding ICD implantation. Given the potential cost saved for de novo ICD implantation as well as (potentially) associated follow-up cost and cost of complications, this strategy may in addition also turn out cost-effective, but comprehensive analyses in this regard are currently lacking.

### Final thoughts

In the year 2015, many interesting studies have surfaced in the field of invasive electrophysiology and cardiac devices, most of which may have (or do already have) important implications for daily clinical practice. Ongoing confirmation and expansion of these data with experience from the real world will be crucial to substantiate their efficacy and safety in the 'real world'. Coverage of all of the exciting developments in one concise review is impossible; as such, various methods and technologies had to be omitted for the time being, including some preliminary results on the use of multi-site pacing and comparisons of point-by-point vs. single shot ablation. If the rate and quality of innovation persists, undoubtedly the year 2016 will equally be a successful one in the field of arrhythmias.
